# Efficient Integration of Rate-Adaptive Reconciliation with Syndrome-Based Error Estimation and Subblock Confirmation for Quantum Key Distribution

**DOI:** 10.3390/e26010053

**Published:** 2024-01-07

**Authors:** Patcharapong Treeviriyanupab, Chun-Mei Zhang

**Affiliations:** 1 Department of Information Technology, Faculty of Science and Technology, Phranakhon Rajabhat University (PNRU), Bangkok 10220, Thailand; 2 Institute of Quantum Information and Technology, Nanjing University of Posts and Telecommunications, Nanjing 210003, China; cmz@njupt.edu.cn

**Keywords:** quantum key distribution, post-processing, low-density parity-check codes, rate-adaptive information reconciliation, syndrome-based error estimation, subblock confirmation

## Abstract

An effective post-processing algorithm is essential for achieving high rates of secret key generation in quantum key distribution. This work introduces an approach to quantum key distribution post-processing by integrating the three main steps into a unified procedure: syndrome-based error estimation, rate-adaptive reconciliation, and subblock confirmation. The proposed scheme employs low-density parity-check codes to estimate the quantum bit error rate using the syndrome information, and to optimize the channel coding rates based on the Slepian–Wolf coding scheme for the rate-adaptive method. Additionally, this scheme incorporates polynomial-based hash verification in the subblock confirmation process. The numerical results show that the syndrome-based estimation significantly enhances the accuracy and consistency of the estimated quantum bit error rate, enabling effective code rate optimization for rate-adaptive reconciliation. The unified approach, which integrates rate-adaptive reconciliation with syndrome-based estimation and subblock confirmation, exhibits superior efficiency, minimizes practical information leakage, reduces communication rounds, and guarantees convergence to the identical key. Furthermore, the simulations indicate that the secret key throughput of this approach achieves the theoretical limit in the context of a BB84 quantum key distribution system.

## 1. Introduction

Cryptography is a well-known technique for achieving communication secrecy, but practical key generation and distribution schemes remain one of the toughest challenges in modern cryptography. The common key distribution protocols [1,2,3] rely on symmetric and asymmetric encryption algorithms, which offer computational security based on the complexity of mathematical problems. However, the security of these protocols could potentially be compromised by the advent of powerful computing devices, such as quantum computers [4,5].

Fortunately, quantum key distribution (QKD) [6] utilizes the properties of quantum mechanics, thereby enabling two legitimate parties (*Alice* and *Bob*) to generate a secret key based on information-theoretic security principles [7]. Basically, the QKD protocol consists of six steps:

**(1)**  **Distribution of quantum information:** In the BB84 protocol [6], *Alice* encodes random bits into the polarization states of single photons, which are then transmitted to *Bob* over the quantum channel. *Bob* subsequently randomly selects measurement bases to measure the polarization of a received photon and obtains classical measurement bits. After this step, both parties have a record of binary information, known as the raw key. Upon accumulating a sufficient amount of raw key, they perform the following post-processing steps [8,9], using the authenticated classical channel to distill secure secret keys.
**(2)**  **Sifting:** *Alice* and *Bob* communicate and compare their encoding and measurement bases. Then, any bits in raw key with non-matching bases are discarded, allowing both parties (*Alice* and *Bob*) to obtain correlated classical bits with the same length, called the sifted key.
**(3)**  **Channel error estimation:** *Alice* and *Bob* typically estimate the quantum bit error rate (QBER) by using random key sampling. If the estimated QBER exceeds a predetermined threshold value, both parties must abort the QKD protocol to prevent potential security breaches.
**(4)**  **Information reconciliation:** *Alice* and *Bob* correct the discrepancies between their sifted keys using error-correction algorithms to produce the reconciled key.
**(5)**  **Confirmation:** *Alice* and *Bob* utilize a universal hash function to verify whether their reconciled keys are identical. If the hash values from *Alice* and *Bob* do not match, they can return to the information reconciliation step or abort the QKD protocol.
**(6)**  **Privacy amplification:** To eliminate any partial information eavesdropped by *Eve* through both the quantum and classical channels, *Alice* and *Bob* compress their identical keys using universal hashing. The resulting shortened keys, known as the secret keys, are statistically independent of *Eve*’s information and are identical between *Alice* and *Bob*.

Although there have been breakthroughs in QKD, the achievable secret key rates remain inadequate for high-speed industrial applications. One of the primary bottlenecks is the efficiency of classical post-processing algorithms. To address this limitation, this work investigates the effective algorithms for error estimation, information reconciliation, and confirmation steps, aiming to enhance the efficiency of classical post-processing.

In QKD post-processing, information reconciliation is typically implemented using interactive protocols such as Cascade [10], which uses a combination of random shuffling and dichotomic search algorithms to identify and correct error positions using subblock parities. Cascade is generally a simple and efficient error-correction protocol. However, its speed is fundamentally limited by its high interactivity. Several modified versions of Cascade have been proposed to improve its speed in parallel operations [11], and to optimize the parameters for optimal implementation [12]. An alternative protocol is Winnow [13], which employs the syndrome from a Hamming code, a type of forward error correction, to reconcile errors in a set of sifted keys while requiring less interactive communication. However, its performance is constrained by the error-correction capabilities of the Hamming code. Additionally, other forward error correction applications, such as Bose–Chaudhuri–Hocquenghem (BCH) [14,15] and low-density parity-check (LDPC) [16,17] codes, have been proposed for the information reconciliation protocol by selecting the appropriate channel coding rates based on a priori QBER estimation. By utilizing irregular LDPC codes and syndrome decoding based on the belief propagation algorithm, the interactive blind reconciliation method [18] can operate without a priori estimation of QBER. This approach relies on interactive communications and continues the reconciliation process until successful decoding is achieved. The technique of blind reconciliation was further adapted with variable step sizes [19] and transformed into a symmetric operation [20], potentially improving efficiency and reducing the required interactivity.

Considering the importance of channel error estimation, the accuracy of a priori error estimation significantly influences the performance of information reconciliation based on LDPC codes. In particular, the value of the estimated QBER is crucial for choosing the optimal LDPC coding rate, which can improve blind reconciliation scenarios by reducing the necessity for additional interactive communications. In [21], the QBER value was estimated using the syndrome information from LDPC codes within a rate-adaptive reconciliation scheme. This syndrome-based QBER estimation approach was further extended in [22] by employing irregular LDPC codes with punctured or shortened bits. Furthermore, the multiple syndrome information obtained from multi-LDPC codes was proposed for error estimation and information reconciliation [23]. In [24], an asymmetric approach to rate-adaptive and blind reconciliation was proposed with a priori error rate estimation based on the exponential moving average of QBER from the previously corrected frames.

Generally, information reconciliation schemes based on LDPC codes cannot always guarantee the exact identity of the reconciled keys due to potential failures in LDPC decoding. These failures can be caused by several factors, such as an insufficient number of iterations during decoding or an inaccurate QBER estimation. Therefore, a confirmation step is necessary to verify the identical key between the two parties. In [25], a confirmation method using a universal hash function was combined with the blind reconciliation. However, this method discards subblocks with mismatched hash values, ensuring only verified identical keys progress to the subsequent step.

Despite considerable progress in information reconciliation based on LDPC codes, most existing methodologies primarily focus on simulations, either as standalone processes [17,18,19,20] or in combination with channel error estimation [21,22,23,24]. To further advance this work, a unified approach is investigated by integrating three main effective algorithms for error estimation, information reconciliation, and confirmation steps. Firstly, the syndrome information generated from the maximum code rate of irregular LDPC codes is employed to determine the QBER value, leveraging the maximum likelihood estimator to cover the potential errors in a QKD system. Then, the estimated QBER value is utilized to the rate-adaptive reconciliation scheme to optimize the appropriate channel coding rate of irregular LDPC codes through puncturing and shortening techniques. Finally, the subblock confirmation using hash verification is seamlessly incorporated with the rate-adaptive reconciliation. This step divides the reconciled keys into subblocks and verifies their identities with a polynomial-based hash function [26]. In contrast to [25], which discards subblocks with mismatched hash values, this approach subjects unverified subblocks to an iterative process of error estimation and information reconciliation. This process continues until all subblocks are successfully verified, thereby guaranteeing that both parties obtain identical keys and avoiding any key discarding. Moreover, the performance of the integrated approach is characterized by various aspects, including the accuracy and the variability in syndrome-based QBER estimation, the information leakage and its correlation with the efficiency metric of information reconciliation, the number of interactive communications, and the success rate of obtaining the identical key. The proposed scheme significantly enhances the efficiency of classical post-processing—achieving a secret key throughput approaching the theoretical limit—compared to other schemes.

The rest of this article is organized as follows. Section 2 reviews the fundamental concepts of information reconciliation and its construction into rate-adaptive reconciliation for QKD post-processing. In Section 3, the efficient integration of syndrome-based error estimation, rate-adaptive reconciliation, and subblock confirmation is presented as a unified procedure. Section 4 presents the comprehensive performance evaluation of the proposed method. Finally, the conclusions and discussions are presented in Section 5.

## 2. Information Reconciliation in QKD Post-Processing

Information reconciliation is a fundamental technique employed in key agreement protocols, involving the extraction of shared information through a public discussion between two correlated sources of random variables. In the context of QKD post-processing, the concepts of channel coding are leveraged to address the information reconciliation problem, based on the Slepian–Wolf coding scheme.

### 2.1. Information Reconciliation Based on Channel Coding Theorem

In the field of communication over classical channels, the channel coding theorem [27] aims to optimize the transmission rate while ensuring reliable communication in the presence of channel noise.

**Figure 1:**
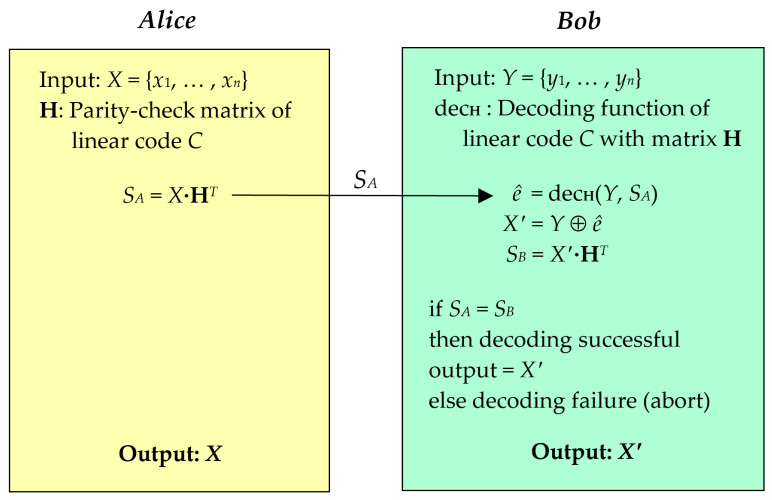
Information reconciliation protocol based on channel coding scheme.

In this section, the problem of information reconciliation is addressed by the concept of the channel coding theorem, as illustrated in Figure 1. Let *C* represent a linear code that contains a parity-check matrix **H** of size *M* × *N*, defined over GF(2) (GF refers to a Galois field). The inputs of the two legitimate parties (*Alice* and *Bob*) are random binary key strings *X* = {*x*_1_, …, *x_n_*} and *Y* = {*y*_1_, …, *y_n_*}, respectively. These inputs, *X* and *Y*, are distributed according to a joint probability distribution *P_XY_* (*x*, *y*), where *x* ∈ *X* and *y* ∈ *Y*. In the context of a QKD system, *P_XY_* (*x*, *y*) can be utilized to determine the discrepancy between *X* and *Y*, which is known as the quantum bit error rate (QBER) value. The simple information reconciliation protocol, which is based on channel coding, enables one-way communication from *Alice* to *Bob* over a classical channel. The protocol can be summarized in the following two main steps:

**(1)**  **Encoding:** *Alice* performs the encoding function of the linear code *C* to generate the syndrome *S_A_*, where *S_A_* = *X*·**H***^T^*. The syndrome *S_A_* is then transmitted over the classical channel to *Bob*.
**(2)**  **Decoding:** Upon receiving the syndrome *S_A_*, *Bob* computes *ê* using the decoding function of *C*, denoted by dec**_H_**, where *ê* = dec**_H_**(*Y*, *S_A_*). The value of *ê* indicates the error position in *Y*, and *Bob* calculates the output value *X*′ by performing *X*′ = *Y* ⊕ *ê*.

The effectiveness of the information reconciliation protocol based on channel coding relies on the capability of the syndrome decoding process to generate an output syndrome *S_B_* = *X*′·**H***^T^* that matches the received syndrome *S_A_* from *Alice* (*S_A_* = *S_B_*). If the decoding process fails, the system must be aborted or reprocessed until successful decoding is achieved.

In this system, the cardinality of *S_A_*, denoted as |*S_A_*|, represents the information leakage, which depends on the correlation of channel capacity in the channel coding scheme. The minimum theoretical information leakage of information reconciliation based on channel coding, represented as *min*(|*S_A_*|), can be calculated as

(1) $$\frac{\min\;(\left|S_{A}\right|)}{N}=H(X|Y),$$

where *N* is the size of input *X*, and *H*(*X*|*Y*) denotes the conditional entropy of *X* given *Y*. However, *Eve* can obtain information leakage when communication occurs over a public channel. Importantly, |*S_A_*| is a fundamental parameter for evaluating the efficiency of the information reconciliation protocol based on channel coding.

### 2.2. Application of Slepian–Wolf Coding to Information Reconciliation Based on Channel Coding

The Slepian–Wolf theorem [28] is a fundamental principle in information theory that addresses the task of conducting effective lossless compression of two correlated data sources. This theorem has significant applications for resolving issues of information reconciliation through channel coding scenarios. In the context of QKD post-processing, the sifted keys of *Alice* and *Bob* are not perfectly correlated; they are represented as binary random variables *X* and *Y*, respectively.

In general, a binary symmetric channel (BSC) is a simplified model that is commonly used to characterize transmission errors in the context of discrete-variable quantum key distribution (DV-QKD) protocols. Therefore, the framework of the information reconciliation protocol based on the channel coding scheme, as illustrated in Figure 1, can be employed within the system of Slepian–Wolf coding. The objective of this framework is to transform the sifted keys *Y* and *X* into a pair of identical keys, such that the reconciled key *X*′ of *Bob* is identical to *X* with probability equal to one, denoted as *P*[*X*′ = *X*] = 1. To achieve this goal, *Bob* requires a certain minimum quantity of syndrome |*S_X_*| from *Alice*. This requirement is defined by the Slepian–Wolf lower bound on the compression rate *R_S_*, which must be at least equal to the conditional entropy *H*(*X*|*Y*), denoted as *R_S_* ≥ *H*(*X*|*Y*).

**Figure 2:**
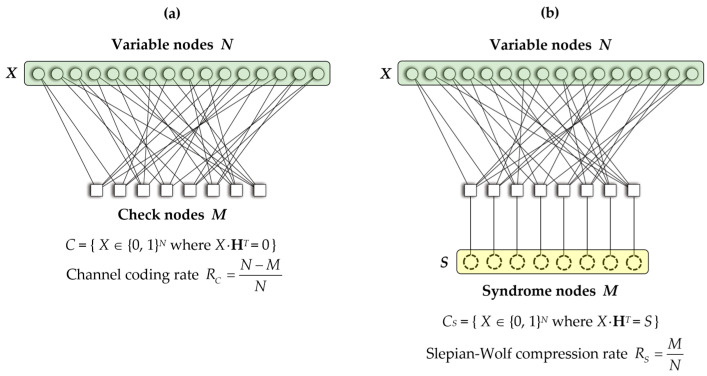
Tanner graph representation of a binary linear block code *C* with a parity-check matrix **H** of dimension *M* × *N*. This graph illustrates the relationship between channel coding and Slepian–Wolf coding: (**a**) Structure of the tanner graph for channel coding, where *N* is the number of variable nodes, *M* is the number of check nodes, and the channel coding rate of linear code (*R_C_*) is $\frac{N-M}{N}$ . (**b**) Structure of the Tanner graph for Slepian–Wolf coding, where *N* is the number of variable nodes, *M* is the number of syndrome nodes, and the compression rate of syndrome (*R_S_*) is $\frac{\;M}{N}$ .

In Figure 2, a simple Tanner graph is shown; it corresponds to a binary linear block code *C* with a parity-check matrix **H** of size *M* × *N*. Generally, the syndrome *S* can be calculated by compressing the main information *X*, where *S* = *X*·**H***^T^*. Correspondingly in Slepian–Wolf coding, the compression rate of the syndrome is represented as $R_{S}=$ $\frac{\;M}{N}$ . This rate is equivalent to the channel coding rate of the linear code *C*, expressed as $R_{C}=\frac{N-M}{N}$ . Therefore, the relationship between Slepian–Wolf compression rate *R_S_* and channel coding rate *R_C_* can be expressed as:

(2) $$R_{S}=1-R_{C}$$

To achieve efficient information reconciliation, the channel coding rate *R_C_* must be optimized to satisfy the Slepian–Wolf lower bound, which is *R_S_* ≥ *H*(*X*|*Y*). Then, it can be rewritten as:

(3) $$1-R_{C}\geq H(X|Y)\mathrm{and}H(X|Y)=H(q),$$

where *q* is the cross-over probability distribution between *X* and *Y* over BSC. In the context of a QKD system, *q* corresponds to the quantum bit error rate (QBER), which quantifies the joint probability distribution among the correlated information from *Alice*, *Bob*, and *Eve*.

### 2.3. Rate-Adaptive LDPC Codes and Efficiency Metric of Information Reconciliation

Low-density parity-check (LDPC) codes [29] are a class of linear block code characterized by their sparse parity-check matrix. Typically, LDPC codes utilize iterative algorithms such as the bit-flipping algorithm, belief propagation algorithm, and min-sum algorithm for the decoding process. According to their structures, LDPC codes can be broadly categorized into regular LDPC codes and irregular LDPC codes. For the regular LDPC codes, each variable node connects to a fixed number of check nodes, which ensures a consistent and fixed number of non-zero entries in each row and column of the parity-check matrix, leading to a uniform degree distribution. Conversely, for the irregular LDPC codes with a non-uniform degree distribution, specific variable nodes can connect to varying numbers of check nodes. The degree distribution of these irregular LDPC codes is designed for specific applications such as wireless communications, digital television broadcasting, and satellite communications. These codes exhibit superior error-correcting capabilities when compared to conventional regular LDPC codes.

For information reconciliation with irregular LDPC codes, a prior error estimation is essential to optimize the channel coding rates, which significantly enhances the efficiency of rate-adaptive information reconciliation. Consider an irregular LDPC code with a parity-check matrix **H** of size *M* × *N*. The mother code rate, denoted as $R_{C}^{\;0}$ , is defined by $R_{C}^{\;0}=\frac{N-M}{N}$ . To fine-tune the LDPC’s mother code rate, puncturing and shortening techniques [30,31] are employed to modulate the optimal coding rate, represented as $R_{C}^{\;(\mathrm{opt})}$ . In this context, *n_p_* and *n_s_* represent the number of punctured and shortened bits within block length *N*, respectively. The equation for $R_{C}^{\;(\mathrm{opt})}$ is given by:

(4) $$R_{C}^{\left(opt\right)}=\frac{N-M-n_{s}}{N-n_{p}-n_{s}}.$$

The theoretically secret key rate after QKD post-processing, denoted as *r_th_*, can be expressed by $r_{\mathrm{th}}=H\left(X|Z\right)\;H\left(X|Y\right)$ . Within the QKD context, *X* and *Y* correspond to the sifted key of *Alice* and *Bob*, respectively, and *Z* represents the information that *Eve* extracts from the quantum channel. The conditional entropy *H*(*X*|*Z*) measures the uncertainty associated with *Eve*’s knowledge about *Alice*’s sifted key. *H*(*X*|*Y*) represents the theoretical information leakage during the information reconciliation phase, which depends on the minimal number of syndrome bits min(|*S_X_*|), as expressed in Equation (1). However, the number of syndrome bits, which corresponds to the Slepian–Wolf compression rate *R_S_*, must exceed the quantity of theoretical information leakage *H*(*X*|*Y*). Therefore, the evaluation of information reconciliation is determined by the ratio of practical information leakage to the theoretical limit, as expressed in the following:

(5) $$\eta_{\;IR}=\frac{R_{S}}{H(X|Y)}=\frac{M}{N\cdot H(q)}\geq 1,$$

where $\eta_{\mathrm{IR}}$ denotes the efficiency metric of information reconciliation, and *H*(*q*) represents the binary entropy function of QBER, which can be calculated as $H(q)=q\log_{2}q\;(1\;q)\log_{2}(1\;q)$ . Considering the lower bound of the Slepian–Wolf compression rate, represented as *R_S_* = *H*(*X*|*Y*), it is noted that $\eta_{\mathrm{IR}}$ must be equal to one to achieve *min*(|*S_A_*|). Notably, the maximum tolerable QBER to ensure information-theoretic security is 11% [32].

For the rate-adaptive reconciliation, the optimal coding rate for LDPC codes $R_{C}^{\left(\mathrm{opt}\right)}$ is modulated using puncturing and shortening techniques, as derived from Equation (4). Therefore, the efficiency of rate-adaptive reconciliation can be determined as a function of the a priori QBER estimation *q_est_*, as follows:

(6) $$\eta_{\;IR}=\frac{1-R_{C}^{\left(opt\right)}}{H(q_{est})}=\frac{M-n_{p}}{\left(N-n_{p}-n_{S})\cdot H(q_{est}\right)}.$$

Furthermore, the secret key throughput ( $\tau_{\mathrm{SK}}$ ) can be used to evaluate the performance of QKD post-processing by considering the inherent parameters of a BB84 QKD system [33,34,35]. It is defined as follows:

(7) $$\tau_{\mathrm{SK}}=p_{exp}\cdot \varepsilon_{\mathrm{BB}84}\cdot f_{clk}\cdot (1-\mathrm{FER})\cdot r_{real},$$

where:

-
$p_{\exp}$ is the total detection rate for events where photons are transmitted from *Alice* to *Bob*.
-
$\varepsilon_{\mathrm{BB}84}$ is the theoretical efficiency of the BB84 protocol.
-
$f_{\mathrm{clk}}$ is the operational clock rate of QKD devices.
- FER is the frame error rate, indicating the failure probability of decoding, which affects the likelihood of non-identical reconciled keys for *Alice* and *Bob*.
-
$r_{\mathrm{real}}$ is the actual secret key rate, depending on $\eta_{\mathrm{IR}}$ from Equation (6), which can be defined as $r_{\mathrm{real}}=H\left(X|Z\right)\;\eta_{\mathrm{IR}}\cdot \;H\left(X|Y\right)$ .

## 3. Rate-Adaptive LDPC Codes for Information Reconciliation: Integrating Syndrome-Based Error Estimation and Subblock Confirmation

In this section, the proposed rate-adaptive LDPC codes are introduced for an information reconciliation protocol and its integration with effective algorithms in channel error estimation and confirmation steps. In the context of QKD post-processing, the proposed scheme commences after the quantum information distribution and sifting steps of the discrete-variable quantum key distribution (DV-QKD) protocol. At this stage, *Alice* and *Bob* obtain correlated sifted keys of the same length, which then proceed to the subsequent steps of the proposed syndrome-based error estimation, rate-adaptive reconciliation, and polynomial-based hash subblock confirmation. 

**Figure 3:**
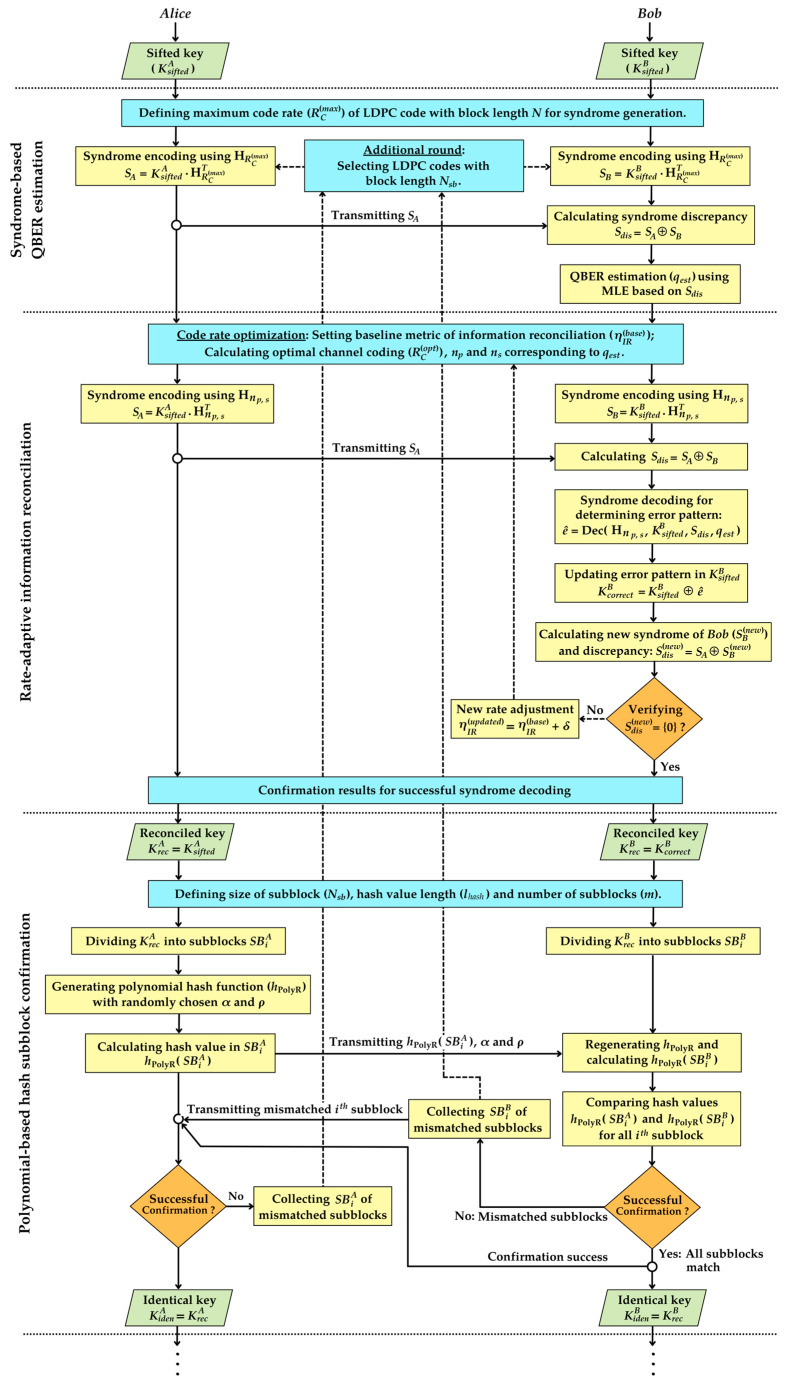
Flowchart of the unified procedure for syndrome-based error estimation, rate-adaptive information reconciliation, and subblock confirmation using polynomial hashing.

Firstly, the syndrome-based error estimation provides the value of the estimated QBER by utilizing the maximum likelihood estimator, which is based on the syndrome encoding of sifted keys between *Alice* and *Bob* using the maximum code rate $R_{C}^{\;(\max)}$ of irregular LDPC codes. The estimated QBER is subsequently used to adjust the optimal channel coding rate $R_{C}^{\;(\mathrm{opt})}$ of irregular LDPC codes. The aim of this adjustment is to optimize $R_{C}^{\;(\mathrm{opt})}$ by determining the number of puncturing bits $n_{p}$ , and the number of shortening bits $n_{s}$ for the rate-adaptive reconciliation scheme. After completing the information reconciliation steps, the reconciled keys are segmented into subblocks. A specific polynomial-based hash function is then randomly generated and subsequently employed for key identity verification within each subblock. If certain subblocks fail verification, the keys from these unverified subblocks are subjected to additional rounds of syndrome-based error estimation and rate-adaptive reconciliation by using irregular LDPC codes with block lengths that match the sizes of the subblocks. This process is repeated until all subblocks achieve successful verification during the confirmation step, which effectively prevents key discarding in QKD post-processing. Figure 3 illustrates the flowchart of the proposed scheme, which includes syndrome-based error estimation, rate-adaptive reconciliation, and polynomial-based hash subblock confirmation. This flowchart is described by the following four main steps:

**(1)**  **Initialization of LDPC code parameters:** *Alice* and *Bob* mutually agree on two sets of irregular LDPC codes with block lengths *N* and $N_{\mathrm{sb}}$ . Specifically, *N* corresponds to the size of the sifted key in the primary round of each post-processing cycle, and $N_{\mathrm{sb}}$ pertains to the size of the subblock in the additional round, employed only after a failure of the confirmation step. Each of these two irregular LDPC codes includes a set of mother code rates, as follows:
(8) $$ℛ=\{R_{C_{1}}^{0},\;\;R_{C_{2}}^{0},\;\;\ldots \;\;,\;\;R_{C_{n}}^{0}\}.$$
  These rates are fine-tuned using puncturing and shortening techniques to select the appropriate code rate for the rate-adaptive information reconciliation step.
**(2)**  **Syndrome-based QBER estimation:** This step utilizes the properties of a maximum likelihood estimator based on the syndrome information of LDPC codes to estimate QBER over the possible ranges of errors in a QKD system. The process consists of the following subsequent steps:
  **(2.1)**  **Syndrome encoding**: *Alice* and *Bob* generate their syndrome information, denoted as *S_A_* and *S_B_*, by encoding their sifted keys $K_{\mathrm{sifted}}^{\;A}$ and $K_{\mathrm{sifted}}^{B}$ , respectively. Both parties employ the syndrome encoding formula ${S_{\left(A\;/\;B\right)}=K}_{\mathrm{sifted}}^{\left(A\;/\;B\right)}\cdot H_{R_{C}^{\left(\max\right)}}^{T}$ , where $H_{R_{C}^{\left(\max\right)}}^{T}$ is the transpose of the parity-check matrix associated with the maximum code rate $R_{C}^{\left(\max\right)}$ with block length *N*. Then, *Alice* transmits *S_A_* to *Bob* over the authenticated classical channel.
  **(2.2)**  **Calculation of syndrome discrepancy**: On *Bob*’s side, the syndrome discrepancy, denoted as *S_dis_*, is determined by calculating the difference between the syndrome information *S_A_* and *S_B_*. Specifically, *S_dis_* = *S_A_* ⊕ *S_B_*, where ⊕ signifies the bitwise XOR operation.
  **(2.3)**  **QBER estimation**: *Bob* computes the initial estimate of QBER (*q_est_*) using a maximum likelihood estimator (MLE) based on *S_dis_* [21,22,23]. The estimation is determined as follows:
(9) $$q_{est}=\underset{q\in [0,\;\;q_{threshold}]}{\arg\max}\;\;L(q|S_{dis}),$$
    where $L(q\;|\;S_{\mathrm{dis}})$ is the likelihood function for estimating the value of QBER *q* based on *S_dis_*, and *q_threshold_* is the maximum QBER threshold that ensures the security of the QKD system. Within this context, each *i*^th^ syndrome bit is probabilistically determined by a Bernoulli distribution. The explicit expression for $L(q\;|\;S_{\mathrm{dis}})$ is given as follows:
(10) $$L(q|S_{dis})=\prod_{i,\;j\;=\;\;1}^{m}\{\begin{array}{ll} p(q,\;\;d_{c}^{\left(i\right)}) & \mathrm{if}\;S_{dis}[i]=1\\ 1-p(q,\;\;d_{c}^{\left(j\right)}) & \mathrm{if}\;S_{dis}[j]=0 \end{array},$$
    where $\mathrm{Pr}(S_{\mathrm{dis}}[i]=1)=p(q,\;\;d_{c}^{\left(i\right)})$ , $\mathrm{Pr}(S_{\mathrm{dis}}[j]=0)=1-p(q,\;\;d_{c}^{\left(j\right)})$ , and $d_{c}^{\;(i)}$ denotes the number of 1s in the *i*^th^ row of the parity-check matrix **H** for irregular LDPC codes. Specifically, when the syndrome bits originate from a mother code with its maximum rate $R_{C}^{\;(\max)}$ , the likelihood function $L(q\;|\;S_{\mathrm{dis}})$ can be reformulated as follows:
(11) $$L(q|S_{dis})=\prod_{i=1}^{m}\left(1-S_{dis}[i]+(2\cdot S_{dis}[i]-1)\cdot p(q,\;\;\;d_{c}^{\left(i\right)})\right),$$
    where the function $p(q,\;d_{c}^{\;(i)})$ , which signifies the probability that *S_dis_*[*i*] = 1, is defined as:
(12) $$p(q,\;\;d_{c}^{\left(i\right)})=\overset{d_{c}^{\left(i\right)}}{\underset{\begin{array}{l} \;\;\;\;\;\;\;\;\;k\;=\;\;1\\ k\;\mathrm{mod}\;\;2\;\;=\;\;1 \end{array}}{\sum }}\left(\begin{array}{c} d_{c}^{\left(i\right)}\\ k \end{array}\right)q^{k}{\left(1-q\right)}^{d_{c}^{\left(i\right)}-\;\;k}.$$
    Furthermore, the syndrome bits are generated from the parity-check matrix, which is affected by the puncturing and shortening positions, denoted as *p* and *s*, respectively. For accurate QBER estimation, it is essential to use only syndrome bits that reflect QBER influences, excluding those adjusted by *p* and *s* at the position *S_dis_*[*i*]. In this context, *ω_i_* denotes the set of positions with entries of 1 in the *i*th row of the parity-check matrix **H**, and essentially identifies the position *S_dis_* [ *i* ]. To mitigate the effects of punctured and shortening, it is necessary to ensure that *ω_i_* ∩ *p* = ∅. The count of bit positions in the *i*th row of matrix **H** corresponding to *ω_i_* is then defined as $d_{C}^{\;(i)}$ = $d_{C}-|\omega_{i}\cap s|$ [22]. Consequently, the likelihood function $L(q\;|\;S_{\mathrm{dis}})$ in Equation (11) can be reformulated as follows:
(13) $$L(q|S_{dis})=\prod_{\begin{array}{l} \;\;\;\;\;\;i=1\;\\ \omega_{i}\cap \;p\;=\;\emptyset \end{array}}^{m}\left(1-S_{dis}[i]+(2\cdot S_{dis}[i]-1)\cdot p(q,\;\;\;d_{c}^{\left(i\right)})\right).$$
    In the syndrome-based QBER estimation, the source of the syndrome information determines the selected likelihood function. If the syndrome information is derived from the original matrix **H** of the mother code $R_{C}^{\;0}$ , the likelihood function $L(q\;|\;S_{\mathrm{dis}})$ , as given in Equation (11), is employed. Conversely, when the syndrome information is generated based on the matrix **H** that incorporates both puncturing and shortening, the likelihood function $L(q\;|\;S_{\mathrm{dis}})$ specified in Equation (13) is considered as the appropriate approach for syndrome-based QBER estimation. After this step, *Bob* obtains the value of the estimated QBER (*q_est_*). This value is then communicated to *Alice* and is subsequently used to determine the optimal coding rate $R_{C}^{\;(\mathrm{opt})}$ in the information reconciliation step. If *q_est_* exceeds the maximum tolerable QBER of 11% [32], the system is required to abort, thereby preventing the use of these sifted keys in subsequent steps to ensure security.
**(3)**  **Rate-adaptive information reconciliation**: In this step, *Alice* and *Bob* employ the value of the estimated QBER (*q_est_*) to optimize the initial coding rate ( $R_{C}^{\;(\mathrm{opt})}$ ). This optimization requires setting up the baseline efficiency metric for information reconciliation ( $\eta_{\mathrm{IR}}^{\left(\mathrm{base}\right)}$ ), which is used to calculate the number of puncturing bits ( $n_{p}$ ) and shortening bits ( $n_{s}$ ) based on *q_est_*, as defined in Equation (6). This metric ensures that *Alice* can generate sufficient syndrome information, allowing *Bob* to decode and correct errors within his sifted key. It is essential to note that $\eta_{\mathrm{IR}}^{\left(\mathrm{base}\right)}$ is obtained from the performance evaluation when deploying the specific irregular LDPC codes in the experimental settings. This process comprises the following subsequent steps:
  **(3.1)**  **Code rate optimization**: *Alice* and *Bob* collaboratively select a set of mother code rates R , associated with the block length *N*. They also agree on the baseline efficiency metric for information reconciliation ( $\eta_{\mathrm{IR}}^{\left(\mathrm{base}\right)}$ ), which is employed to calculate the optimal coding rate ( $R_{C}^{\left(\mathrm{opt}\right)}$ ) based on the entropy function of the estimated QBER ( $H(q_{\mathrm{est}})$ ). In this scenario, the mother code rate ( $R_{C}^{\;0})$ is selected from R such that its value is closest to the calculated $R_{C}^{\;(\mathrm{opt})}$ . According to Equation (6), $R_{C}^{\;(\mathrm{opt})}$ is derived using:
(14) $$R_{C}^{\left(opt\right)}=1-[\eta_{\;IR}\cdot H(q_{est})].$$
    To identify the desired $R_{C}^{\;0}$ from the set R , the selection criteria is determined by
(15) $$R_{C}^{0}=\underset{R\in ℛ}{\mathrm{argmin}\;\;}\left|R-R_{C}^{\left(\mathrm{opt}\right)}\right|,$$
    where $R_{C}^{\;0}=\frac{N-M}{N}$ . After selection, $R_{C}^{\;0}$ is then adapted to attain the value of $R_{C}^{\;(\mathrm{opt})}$ by adjusting the parameters of $n_{p}$ and $n_{s}$ , given as Equation (4). Importantly, the relationship between the puncturing and shortening parameters is defined by $n_{d}$ = $n_{p}$ + $n_{s}$ , where $n_{d}$ is the total number of punctured and shortened bits used to determine the values for $n_{p}$ and $n_{s}$ . Consequently, both $n_{p}$ and $n_{s}$ can be derived from Equation (6) by considering $\eta_{\mathrm{IR}}^{\left(\mathrm{base}\right)}$ and $H(q_{\mathrm{est}}),$ as
(16) $$n_{p}=\left\lceil M-[(N-n_{d})\cdot \eta_{IR}^{\left(base\right)}\cdot H(q_{est})]\right\rceil ;\;n_{s}=n_{d}-n_{p},$$
    where ⌈…⌉ is the ceiling function rounding $n_{p}$ up to the nearest integer, and both $n_{p}$ and $n_{s}$ must be the positive value. If either $n_{p}$ or $n_{s}$ is calculated to be negative, the selection of the mother code rate is decreased to the next available $R_{C}^{\;0}$ in the set R . The values of $n_{p}$ and $n_{s}$ are then recalculated using this newly selected $R_{C}^{\;0}$ according to Equation (16), ensuring both $n_{p}$ and $n_{s}$ are positive. After successfully determining $n_{p}$ and $n_{s}$ , specific positions for puncturing (*p*) and shortening (*s*) are identified and used to modify the original parity-check matrix **H** of the selected $R_{C}^{\;0}$ . The modified matrix ( $H_{n_{p,s}}$ ) is then employed in subsequent syndrome encoding and decoding processes.
  **(3.2)**  **Syndrome encoding**: *Alice* and *Bob* employ the modified matrix ( $H_{n_{p,s}}$ ) of $R_{C}^{\;(\mathrm{opt})}$ to encode their sifted keys $K_{\mathrm{sifted}}^{\;A}$ and $K_{\mathrm{sifted}}^{B}$ , respectively. Both parties apply the syndrome encoding ${S_{\left(A\;/\;B\right)}=K}_{\mathrm{sifted}}^{\left(A\;/\;B\right)}\cdot H_{n_{p,s}}$ to produce the appropriate amount of syndrome information based on *q_est_*. Subsequently, *Alice* sends *S_A_* to *Bob* through the authenticated classical channel.
  **(3.3)**  **Syndrome decoding and verification**: In the syndrome decoding step, *Bob* utilizes the syndrome information *S_A_* received from *Alice* and his syndrome *S_B_* to compute the discrepancy syndrome *S_dis_* = *S_A_* ⊕ *S_B_*. The decoding process is performed by the belief propagation algorithm, which employs the log-likelihood ratios to identify the error pattern $\hat{e}$ within the sifted key $K_{\mathrm{sifted}}^{B}$ . This algorithm operates with the modified parity-check matrix ( $H_{n_{p,s}}$ ), which corresponds to the specific positions of puncturing *p* and shortening *s*. Within this context, the value of QBER estimation (*q_est_*) is utilized to model the transmission errors as the crossover probability in a binary symmetric channel (BSC). The simplified decoding function for determining the error pattern $\hat{e}$ is expressed as:
(17) $$\hat{e}=\mathrm{Dec}(H_{n_{p,\;s}},\;\;K_{sifted}^{B},\;\;S_{dis},\;\;q_{est}).$$
    Then, *Bob* uses $\hat{e}$ to update $K_{\mathrm{sifted}}^{B}$ , resulting in $K_{\mathrm{correct}}^{B}=K_{\mathrm{sifted}}^{B}⊕\hat{e}$ . To verify the success of syndrome decoding, the proposed scheme introduces a step that re-checks the syndrome discrepancy. By employing the same parity-check matrix $H_{n_{p,s}}$ , *Bob* computes the new syndrome $S_{B}^{\;(\mathrm{new})}$ and then calculates the new syndrome discrepancy with *S_A_*, expressed as $S_{\mathrm{dis}}^{\;(\mathrm{new})}=S_{A}⊕S_{B}^{\left(\mathrm{new}\right)}$ . If $S_{\mathrm{dis}}^{\;(\mathrm{new})}=\left\{0\right\}$ , it confirms successful decoding between *Alice* and *Bob*. Otherwise, a decoding failure feedback is announced, and the protocol returns to step 3.1 to adjust the new code rate ( $R_{C}^{\;(\mathrm{new})}$ ). In this case, the initial metric for information reconciliation is incremented by the factor *δ*, expressed as $\eta_{\mathrm{IR}}^{\left(\mathrm{updated}\right)}$ = $\eta_{\mathrm{IR}}^{\left(\mathrm{base}\right)}$ + *δ*. This updated $\eta_{\mathrm{IR}}^{\left(\mathrm{updated}\right)}$ is subsequently employed to determine the new values for $n_{p}$ and $n_{s}$ with respect to $R_{C}^{\;(\mathrm{new})}$ . The adjusted rate $R_{C}^{\;(\mathrm{new})}$ is then applied in the re-processing of syndrome encoding and decoding. After the successful decoding, both *Alice* and *Bob* exclude the bit positions that are affected by puncturing and shortening, as represented by *p* ∪ *s*.
**(4)**  **Polynomial-based hash subblock confirmation**: In the confirmation step, a polynomial-based hash function [26], a form of universal hashing, is employed to verify the equality of *Alice* and *Bob*’s reconciled keys $K_{\mathrm{rec}}^{A}$ and $K_{\mathrm{rec}}^{B}$ . To mitigate the risk of discarding the entire key due to a confirmation failure, both parties adopt a subblock verification approach by partitioning the reconciled keys into subblocks of size $N_{\mathrm{sb}}$ . Subsequently, a polynomial hash value is generated for each subblock to verify its integrity. This process is divided into the following steps:
  **(4.1)**  **Dividing the reconciled keys into subblocks**: *Alice* and *Bob* update the sizes of their reconciled keys $N_{\mathrm{rec}}$ and then divide $K_{\mathrm{rec}}^{A}$ and $K_{\mathrm{rec}}^{B}$ into subblocks of size $N_{\mathrm{sb}}$ . Each subblock is referred to as the *i^th^* subblock, where *i* ranges from 1 to *m*, and $m=N/{\;N}_{\mathrm{sb}}$ . The partition of the reconciled keys in each subblock of *Alice* and *Bob* ( ${\mathrm{SB}}_{\;i}^{\left(A/B\right)}$ ) is defined as:
(18) $$SB_{i}^{\left(A/B\right)}=\left\{k_{(i-1)\;\;\times \;\;N_{sb}+1}^{\left(A/B\right)},\;\;k_{(i-1)\;\;\times \;\;N_{sb}+2}^{\left(A/B\right)},\;\;\ldots \;\;,\;\;k_{i\;\;\times \;\;N_{sb}}^{\left(A/B\right)}\right\}.$$
    Afterward, *Alice* and *Bob* have corresponding subblocks of their reconciled keys, denoted as ${\mathrm{SB}}_{\;i}^{\;A}$ and ${\mathrm{SB}}_{\;i}^{\;B}$ , respectively.
  **(4.2)**  **Generation of polynomial hash function and hash values calculation**: To generate the polynomial hash function, *Alice* first defines the hash value length ( $l_{\mathrm{hash}})$ and then randomly selects the parameters of the polynomial base α and the prime modulus ρ . Specifically, α is chosen from the set α ∈ {2, 3, …, ρ−2 }, and ρ is a prime number constrained by $\rho <2^{l_{\mathrm{hash}}}$ . Subsequently, the polynomial hash function is applied to calculate the hash values of the reconciled keys in each subblock ${\mathrm{SB}}_{\;i}^{\;A}$ . This can be mathematically represented as
(19) $$h_{\;\mathrm{PolyR}}(SB_{i}^{A})=\left(k_{0}^{A}+\;\;k_{1}^{A}\cdot \alpha \;\;+\;\;k_{2}^{A}\cdot \alpha^{2}+\;\;\cdots \;\;+\;\;k_{N_{sb}-1}^{A}\cdot \alpha^{N_{sb}-1}\right)\;\;\;\;\mathrm{mod}\;\rho ,$$
    Following this calculation, *Alice* transmits the resulting hash values for each subblock $h_{\mathrm{PolyR}}({\mathrm{SB}}_{\;i}^{\;A}$ ) to *Bob*. This transmission corresponds to the parameters of the polynomial hash function α and ρ , which were previously chosen by *Alice*.
  **(4.3)**  **Hash verification and result confirmation**: On *Bob*’s side, he uses the received parameters α and ρ to generate the polynomial hash function and computes the corresponding hash values for his reconciled keys in each subblock $h_{\mathrm{PolyR}}({\mathrm{SB}}_{\;i}^{\;B}$ ), as defined in Equation (19). To verify the identical keys, *Bob* compares $h_{\mathrm{PolyR}}({\mathrm{SB}}_{\;i}^{\;B}$ ) with the received $h_{\mathrm{PolyR}}({\mathrm{SB}}_{\;i}^{\;A}$ ) for each subblock. This step is considered successfully completed if the hash values match for all subblocks from 1 to *m*, which is expressed as:
(20) $$\forall i\in \{1\;,\;\;2\;,\;\;\ldots \;\;,\;\;m\}:h_{\mathrm{PolyR}}(SB_{i}^{A})=h_{\mathrm{PolyR}}(SB_{i}^{B}).$$
    Otherwise, *Bob* sends feedback to *Alice* indicating the confirmation failure and identifying the mismatched *i^th^* subblock. Then, only the reconciled keys from the mismatched subblocks are reprocessed, and the procedure returns to the steps of syndrome-based error estimation and rate-adaptive reconciliation in the additional round. During this round, specific irregular LDPC codes with block lengths $N_{\mathrm{sb}}$ are employed to estimate QBER, optimize the code rate, and correct errors using the same procedure. The process continues until the subblocks are successfully verified using polynomial-based hashing. Ultimately, *Alice* and *Bob* obtain the identical keys, denoted as $K_{\mathrm{iden}}^{A}$ and $K_{\mathrm{iden}}^{B}$ , respectively.

## 4. Simulation and Results

In this section, the simulation approach of this work is presented to evaluate the efficiency of the three main schemes: syndrome-based error estimation, rate-adaptive information reconciliation, and polynomial-based hash subblock confirmation. All the initial sifted keys used in this experiment were generated by a pseudo-random number generator (PRNG) based on the observed error rates in a QKD system. The parameters employed for the proposed schemes in this approach are presented in Table 1.

**Figure 4:**
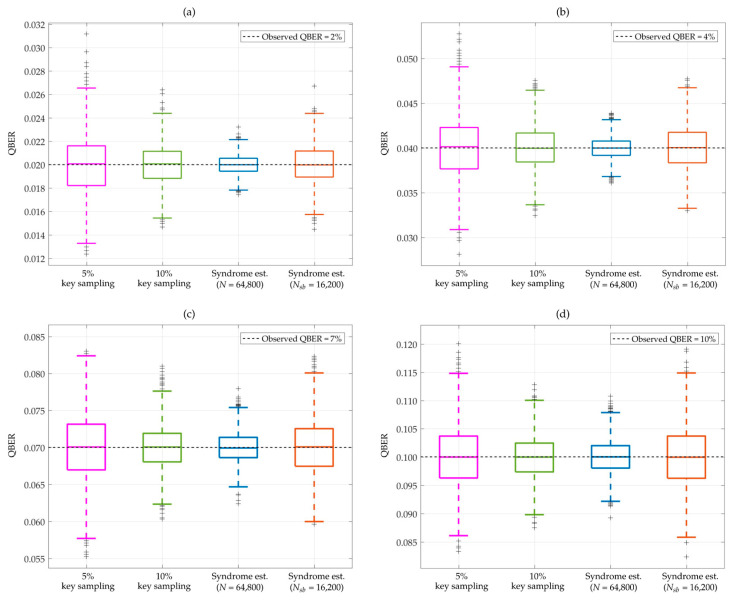
Comparison of QBER estimation methods: random key sampling with 5% and 10% sampling rates from the sifted keys and the syndrome estimation using a block length of *N* = 64,800 bits with $R_{C}^{\left(\max\right)}$ = 9/10 and $N_{\mathrm{sb}}$ = 16,200 bits with $R_{C}^{\left(\max\right)}$ = 8/9. These results are presented using box plots at four distinct observed QBER values: (**a**) 2%, (**b**) 4%, (**c**) 7%, and (**d**) 10%. For each observed QBER value, the results of all QBER estimation methods were derived from 2000 iterations.

First, the performance of the syndrome-based QBER estimation is presented to demonstrate its efficiency in channel error estimation. In this step, the syndrome information from both legitimate parties is derived using the maximum code rate $R_{C}^{\;(\max)}$ . This rate signifies the highest value within the set of mother code rates R . In the proposed scheme, the irregular LDPC codes with two specific block lengths were implemented for syndrome-based QBER estimation. Specifically, the primary round used the block length of *N* = 64,800 bits with $R_{C}^{\;(\max)}$ = 9/10, while the additional rounds employed the block length of $N_{\mathrm{sb}}$ = 16,200 bits with $R_{C}^{\;(\max)}$ = 8/9. Notably, the syndrome information was generated from the sifted keys of both *Alice* and *Bob* using the original parity-check **H** of irregular LDPC codes. This syndrome encoding was operated without any adjustments to the code rate, effectively bypassing the puncturing and shortening bits. Consequently, the value of the quantum bit error rate (QBER) was estimated by the maximum-likelihood estimator, as defined in Equation (11). Based on this parameter setup, Figure 4 presents the comparison results of the proposed syndrome-based QBER estimation and the traditional key sampling method with 5% and 10% sampling rates of the sifted keys for QBER estimation. These numerical results are depicted in box plots, which are derived from 2000 iterations, with the observed QBER set at four distinct values: 2%, 4%, 7%, and 10%. The specific values from these box plots are elaborated in Table 2, which details the performance and underlying statistical distribution of each QBER estimation method.

In Table 2, the mean squared error (MSE) for all observed QBER values (*q_obs_*) are calculated using:

(21) $$\mathrm{Mean}\;\mathrm{accuracy}\;(\%)\;\;=\;\;\frac{1}{n}\sum_{i=1}^{n}\left[(1-\left|q_{est_{\;i}}-q_{obs_{\;i}}\right|\right)\;\;\times \;\;100],$$

and

(22) $$\mathrm{Mean}\;\mathrm{squared}\;\mathrm{error}\;\left(\mathrm{MSE}\right)\;\;=\;\;\frac{1}{n}\sum_{i=1}^{n}{\left(\;q_{est_{\;i}}-q_{obs_{\;i}}\right)}^{\;2},$$

where *n* denotes the number of iterations. 

Based on the performance and statistical metrics presented in Figure 4 and Table 2, the proposed syndrome-based QBER estimation with *N* = 64,800 bits demonstrates the highest accuracy and the smallest mean squared error (MSE) for all observed QBER values (*q_obs_*). Additionally, it exhibits the smallest interquartile range (IQR), with the outlier points closer to the mean value, outperforming the traditional key sampling method. This indicates its ability to handle consistent data distributions, ensuring a compact spread around the median of the estimated QBER results.

In a practical QKD system, even in the absence of an eavesdropper (*Eve*) on the quantum channel, the observed QBER after quantum information distribution is not a consistent value. In this case, the error estimation method must achieve high accuracy to ensure reliable operations, even with varying observed error rates in each QKD cycle. 

**Figure 5:**
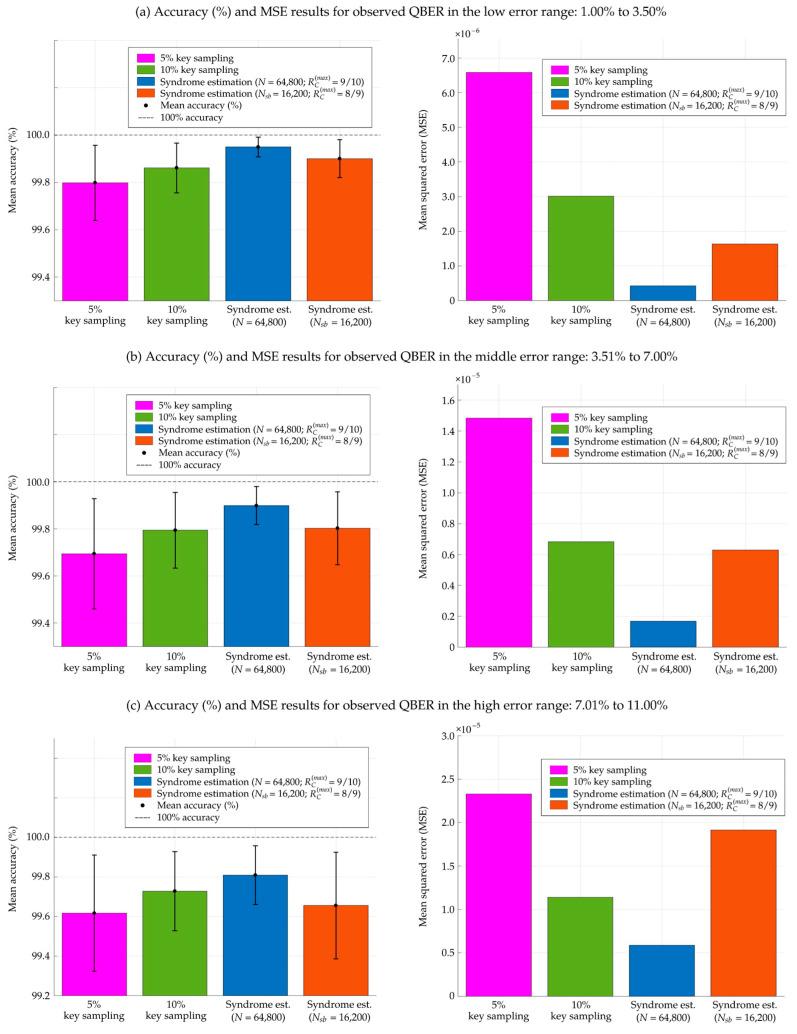
Comparison of various QBER estimation methods in terms of the mean accuracy (%) and the mean squared error (MSE) of estimated QBER (*q_est_*) from 2000 iterations. The comparison of QBER estimation methods encompasses random key sampling with 5% and 10% sampling rates, as well as the syndrome estimation with a block length of *N* = 64,800 bits and $N_{\mathrm{sb}}$ = 16,200 bits. These results are obtained from simulations over three observed QBER ranges: (**a**) low error rates of 1.00–3.50%, (**b**) middle error rates of 3.51–7.00%, and (**c**) high error rates of 7.01–11%.

Figure 5 and Table 3 present the corresponding performance of various QBER estimation methods by considering the variability of a QKD system over three observed QBER ranges—low (1.00–3.50%), middle (3.51–7.00%), and high (7.01–11.00%) error rates—averaged over 2000 iterations. The proposed syndrome-based QBER estimation with a block length of *N* = 64,800 bits consistently performs better than the random key sampling methods over the observed QBER ranges from low to high error rates. As illustrated in Figure 5 and Table 3, this approach achieves the highest mean accuracy (%) and the lowest variability of estimated QBER, as evidenced by its minimal standard deviation (SD). Moreover, its low mean squared error (MSE) further indicates a superior correspondence between the observed and estimated QBER values. While traditional random key sampling serves as a practical and straightforward method for channel error estimation, it must discard a portion of the sifted key that is disclosed for QBER estimation, depending on the sampling rate. In contrast, the syndrome-based QBER estimation method avoids discarding sample keys, thereby preserving the entire size of the sifted keys after channel error estimation for inputting to the subsequent information reconciliation step. However, the amount of syndrome information revealed during the syndrome-based estimation constitutes a portion of the information leakages. This portion is also considered a subset of the syndrome information used in the proposed rate-adaptive reconciliation. These leakages are subsequently mitigated during the privacy amplification step.

Although the syndrome-based QBER estimation using the subblock length of $N_{\mathrm{sb}}$ = 16,200 bits does not outperform the 10% random key estimation in terms of mean accuracy and MSE within the high error range of 7.01–11.00%, as shown in Figure 5 and Table 3, it still achieves notable efficacy with a mean accuracy of 99.6556% and relatively low MSE of 1.9135 × 10^−5^. These results affirm its capability for accurate error estimation in the variability of observed error rates. Crucially, the maximum QBER threshold *q_threshold_* for the syndrome-based estimation was set at 0.25. This threshold corresponds to the maximum QBER typically encountered during an intercept-and-resend attack in the BB84 QKD protocol. Additionally, this experimental setting ensures that the estimated QBER maintains an accuracy above 95%. Specifically, at the observed QBER of 0.25, the syndrome-based estimation with *N* = 64,800 and $N_{\mathrm{sb}}$ = 16,200 bits achieves mean accuracies of 95.1979% and 95.1631%, respectively. However, if the estimated value of QBER exceeds 11% [32] or cannot be determined within *q_threshold_*, the set of sifted keys from this post-processing cycle must be aborted to ensure information-theoretic security in the QKD system. For implementation, the proposed syndrome-based estimation, employing *N* = 64,800 bits and $R_{C}^{\;(\max)}$ = 9/10, is appropriate for the primary round. Meanwhile, $N_{\mathrm{sb}}$ = 16,200 bits with $R_{C}^{\;(\max)}$ = 8/9 can be utilized in the occasional additional rounds. 

To analyze the efficiency of the proposed rate-adaptive reconciliation integrated with syndrome-based QBER estimation and subblock confirmation, the number of information leakages during the information reconciliation step ( $L_{\mathrm{rec}}$ ) is considered to evaluate the efficiency metric of information reconciliation ( $\eta_{\mathrm{IR}}$ ). According to Equation (6), $\eta_{\mathrm{IR}}$ can be reformulated in terms of $L_{\mathrm{rec}}$ , expressed as follows:

(23) $$\eta_{IR}=\frac{L_{rec}}{H(q_{est})\cdot N},$$

where *N* represents the sifted key size. For the proposed scheme, the number of information leakages is quantified by the total amount of syndrome information that *Alice* transmits to *Bob* during the syndrome-based estimation and rate-adaptive reconciliation steps, which consists of two parts. Firstly, it includes the syndrome information derived from the primary round, which handles the full size of the sifted key at *N* = 64,800 bits for the LDPC’s block length. The second part consists of the syndrome information generated during the additional rounds, which were conducted specifically in response to failures in the subblock confirmation step. Each of these unsuccessful subblocks was processed using the specific LDPC codes with block lengths of $N_{\mathrm{sb}}$ = 16,200 bits for syndrome-based estimation and rate-adaptive reconciliation. Therefore, the information leakages of the proposed scheme ( $L_{\mathrm{rec}}$ ) can be formulated by:

(24) $$L_{rec}=\left[M_{R_{C}^{\left(max\right)}}+\;\;\;(R_{C}^{\left(max\right)}-R_{C}^{\left(opt\right)})\cdot N\right]\;\;\;+\;\;\sum_{i=1}^{m_{fail}}\left[M_{sb_{i},\;\;R_{C}^{\left(max\right)}}+\;\;(R_{C,\;\;sb_{i}}^{\left(max\right)}-R_{C,\;\;sb_{i}}^{\left(opt\right)})\cdot N_{sb}\right].$$

In Equation (24), $M_{R_{C}^{\left(\max\right)}}$ represents the number of check nodes in the parity-check matrix $H_{R_{C}^{\left(\max\right)}}$ associated with the maximum code rate $R_{C}^{\;(\max)}$ for syndrome-based estimation. Additionally, $R_{C}^{\;(\mathrm{opt})}$ denotes the calculated optimal coding rate based on the number of puncturing bits $n_{p}$ and shortening bits $n_{s}$ for rate-adaptive reconciliation used in the primary round, as detailed in Equations (14)–(16). Meanwhile, the unverified subblocks from *i* to *m_fai_* are processed in the additional rounds. In this context, ${\;M}_{{\mathrm{sb}}_{i},\;R_{C}^{\left(\max\right)}}$ denotes the number of check nodes in $H_{R_{C}^{\left(\max\right)}}$ associated with subblock lengths $N_{\mathrm{sb}}$ of $R_{C,\;{\mathrm{sb}}_{i}}^{\;(\max)}$ , which is used for syndrome-based estimation; the calculated optimal coding rate $R_{C,\;{\mathrm{sb}}_{i}}^{\;(\mathrm{opt})}$ is used for rate-adaptive reconciliation. In the case of a successful confirmation within the primary round, $L_{\mathrm{rec}}$ relies exclusively on the syndrome information obtained from the primary round. Consequently, additional rounds become unnecessary, and *m_fail_* = 0.

**Figure 6:**
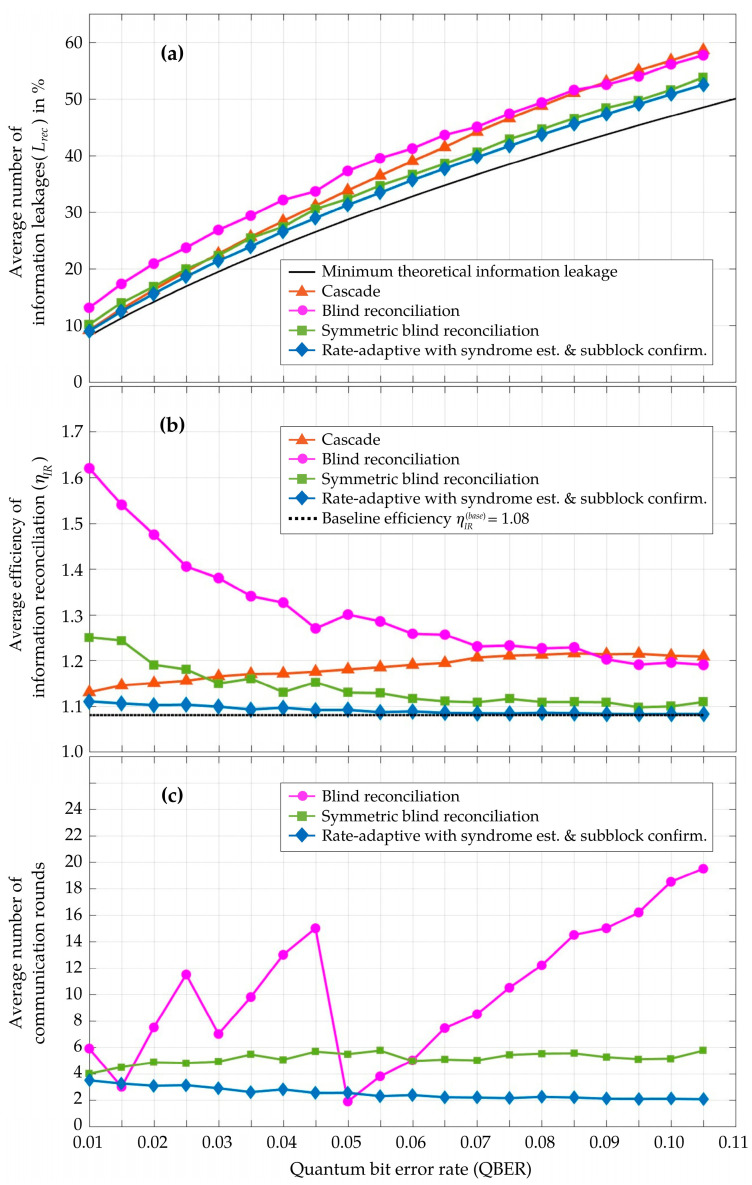
Performance comparison of (**a**) the number of information leakages (*L_rec_*), represented as a percentage; (**b**) the efficiency metric of information reconciliation ( $\eta_{\mathrm{IR}}$); and (**c**) the number of communication rounds during the information reconciliation step. In these simulations, the proposed rate-adaptive reconciliation with syndrome-based QBER estimation and subblock confirmation is evaluated and compared to other methods, including Cascade, blind, and symmetric blind. The simulation results are obtained from an average of 2000 iterations for every observed QBER point.

In Figure 6, the performance of the proposed rate-adaptive reconciliation, integrated with syndrome-based QBER estimation and subblock confirmation, is compared against the existing methods. These methods include Cascade with a frame length of 10^4^ bits [12], blind reconciliation using LDPC codes with a block length of 10^4^ bits [18], and symmetric blind reconciliation with LDPC block lengths of 4 × 10^3^ bits [20]. The comparisons are conducted based on three important evaluation parameters: the number of information leakages $L_{\mathrm{rec}}$ (%), the efficiency metric of information reconciliation $\eta_{\mathrm{IR}}$ , and the number of communication rounds required during the information reconciliation step.

For every observed QBER value, the proposed scheme generates the total amount of syndrome information in both the primary and additional rounds, leading to minimal practical information leakages $L_{\mathrm{rec}}$ during the information reconciliation step. As depicted in Figure 6a, $L_{\mathrm{rec}}$ of the proposed scheme closely approaches the theoretical limit. These information leakages influence the efficiency metric of information reconciliation ( $\eta_{\mathrm{IR}}$ ), as detailed in Equation (23). The proposed scheme also achieves an efficiency metric $\eta_{\mathrm{IR}}$ closer to the perfect information reconciliation ( $\eta_{\mathrm{IR}}$ = 1) than the other methods, as illustrated in Figure 6b. Due to the superior accuracy and minimal variability in QBER estimation with the syndrome-based method, the proposed scheme effectively determines and adapts the optimal code rate $R_{C}^{\;(\mathrm{opt})}$ . Consequently, it requires fewer communication rounds during both the syndrome-based estimation and the rate-adaptive reconciliation processes, compared to both blind and symmetric blind reconciliation methods, as depicted in Figure 6c. However, the interactive reconciliation in both blind and symmetric blind methods, which can operate without a priori QBER estimation, typically require more communication rounds to ensure successful decoding. In the case of Cascade, an average of more than 40 communication rounds are required for a sifted key frame of 10^4^ bits [12] to reconcile the error bits using the dichotomic search algorithm. 

**Figure 7:**
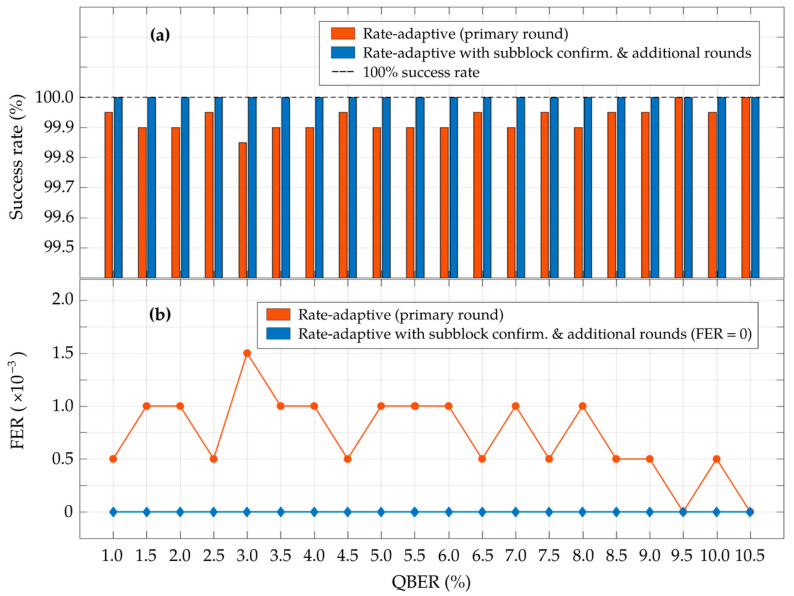
Comparison of (**a**) the success rate (%) and (**b**) the frame error rate (FER) between the proposed rate-adaptive reconciliation performed solely in the primary round, and its integration with subblock confirmation through an iterative process in the additional rounds. These simulation results are obtained from 2000 iterations for every observed QBER value.

Figure 7 presents the success rate and frame error rate (FER) of the proposed scheme, based on the simulation results of 2000 iterations for each observed QBER point. In the primary round (which adopts LDPC codes with block lengths of 64,800 bits), the rate-adaptive reconciliation achieves an average success rate of approximately 99.93% and exhibits an average FER of 7.25 × 10^–4^ for the entire observed QBER range. However, relying solely on the primary round of the rate-adaptive reconciliation does not guarantee the integrity of the identical key between the two legitimate parties. Consequently, the proposed scheme integrated rate-adaptive reconciliation with the subblock confirmation, where the reconciled key from the primary round was divided into subblocks of size ${\;N}_{\mathrm{sb}}$ = 16,200 bits. Subsequently, polynomial-based hashing was employed to detect unverified subblocks, which were then processed in the additional rounds using the syndrome-based estimation and rate-adaptive reconciliation with LDPC codes of block length $N_{\mathrm{sb}}$ . After the completion of the primary and additional rounds, the simulation results of the proposed scheme demonstrate a 100% success rate with an FER of zero for the 2000 iterations, as illustrated in Figure 7. These results guarantee a 100% convergence probability when the proposed rate-adaptive reconciliation is integrated with syndrome-based error estimation and subblock confirmation within this unified procedure. Given this setup, the bound on the collision probability ( $P_{\mathrm{Collision}}$ ) for subblock confirmation with a hash value length $l_{\mathrm{hash}}$ of 64 bits and a subblock $N_{\mathrm{sb}}$ of 16,200 bits is $P_{\mathrm{Collision}}\leq$ 7.11 × 10^−12^ per subblock.

**Figure 8:**
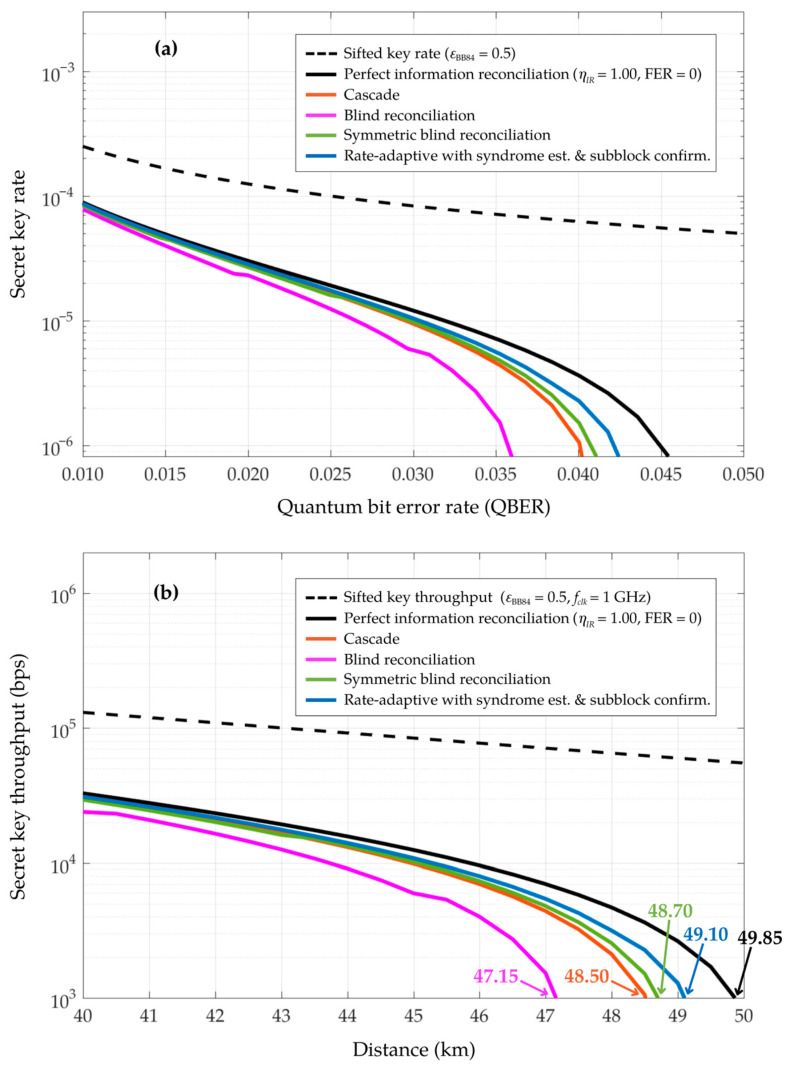
Comparison of (**a**) the secret key rate as a function of the quantum bit error rate (QBER) and (**b**) the secret key throughput as a function of the distance over the quantum channel (km) with a QKD system operating at 1 GHz clock rate, utilizing the parameters of a BB84 QKD system as defined in Table 4. In these simulations, the proposed rate-adaptive reconciliation with syndrome-based estimation and subblock confirmation is evaluated and compared with Cascade, blind, and symmetric blind reconciliation.

In Figure 8, the performance of the proposed rate-adaptive reconciliation with syndrome-based QBER estimation and subblock confirmation is presented by simulating (a) the secret key rate as a function of the quantum bit error rate (QBER) and (b) the secret key throughput as a function of the distance (km) over the quantum channel for a QKD system operating at a 1 GHz clock rate. These results incorporate the inherent properties of the single photon source and detection, as well as the optical fiber losses of the quantum channel for a BB84 QKD protocol, using the parameters listed in Table 4. The proposed scheme achieves the secret key rate and throughput, approaching the theoretical limit of the perfect information reconciliation, where $\eta_{\mathrm{IR}}$ = 1 and FER = 0. It outperforms other methods, such as Cascade, blind reconciliation, and symmetric blind reconciliation. The curve representing perfect information reconciliation, as depicted in Figure 8b, indicates a drop in secret key throughput to 1 Kbps at a transmission distance of approximately 49.85 km. In comparison, the curve for the proposed rate-adaptive reconciliation with integrated syndrome-based estimation and subblock confirmation achieves a throughput of 1 Kbps at a distance of approximately 49.10 km, closely approaching the ideal performance of perfect information reconciliation. Meanwhile, symmetric blind reconciliation, Cascade, and blind reconciliation reach their maximum distances for a throughput of 1 Kbps at approximately 48.70 km, 48.50 km, and 47.15 km, respectively. From these performance evaluations, the unified approach of the proposed scheme significantly improves the achievability of higher secret key throughput over longer transmission distances.

## 5. Conclusions and Discussions

This work investigates an effective approach to QKD post-processing algorithms by focusing on rate-adaptive reconciliation and its integration with syndrome-based error estimation and polynomial-based hash subblock confirmation. By utilizing syndrome-based estimation, this approach significantly improves the accuracy and minimizes the variability of the estimated QBER value. It enables rate-adaptive reconciliation to effectively determine the optimal code rate, consequently reducing the number of communication rounds in practice. Simulation results demonstrate that this unified approach requires fewer information leakages, improves the reconciliation efficiency, and ensures the integrity of identical keys. These findings clearly indicate that the proposed approach can greatly enhance the efficiency of classical post-processing, achieving higher secret key throughput over longer transmission distances.

In QKD post-processing, the amount of information leakage during information reconciliation significantly influences the efficiency of classical post-processing. These leakages are subsequently eliminated in the privacy amplification, which directly reduces the final secret key size, according to the information-theoretic security principles. Efficient information reconciliation based on irregular LDPC codes with large block lengths of up to 10^5^ bits offers superior error-correction capability. This allows for setting the baseline efficiency metric close to the theoretical limit, thereby minimizing the generation of syndrome information, which constitutes the information leakage in practice. Furthermore, the use of an extensive amount of punctured and shortened bits can degrade the error-correction capability of the original code design. To address this issue, this work employs the standard LDPC codes from [36,37] for a set of original code rates, arranged in a sequence from a high to low rate, with each rate closely spaced. This approach minimized the total number of puncturing and shortening bits, enabling efficient adaptation of the original code rate while maintaining robust error-correction capability for realizing the optimal efficiency of classical post-processing.

In future work, the unified approach of the proposed schemes will be analyzed within the context of finite key security to comprehensively assess the overall security parameters. Additionally, this approach will be applied to practical QKD systems, enabling higher-speed QKD applications.

## Figures and Tables

**Table 1:** Parameter setup for the experimental approach used in the proposed schemes: syndrome-based error estimation, rate-adaptive information reconciliation, and subblock confirmation.

Procedure Step	Parameter	Value
Syndrome-based error estimation	Sifted key size (primary/additional round) [bit]	64,800/16,200
	Maximum code rate ( $R_{C}^{\;(\max)}$ )	9/10 for block length *N* 8/9 for block length $N_{\mathrm{sb}}$
	Maximum QBER threshold (*q_threshold_*)	0.25
Rate-adaptive information reconciliation	Block length of LDPC codes: *N* for primary round/ $N_{\mathrm{sb}}$ for additional round [bit]	*N* = 64,800/ $N_{\mathrm{sb}}$ = 16,200
	Set of mother code rates ( R )	For block length *N*: {9/10, 8/9, 154/180, 5/6, 4/5, 7/9, 3/4, 22/30, 128/180, 25/36, 2/3, 116/180, 28/45, 3/5, 26/45, 11/20, 96/180, 1/2} [36,37] For block length $N_{\mathrm{sb}}$ : {8/9, 5/6, 4/5, 3/4, 32/45, 2/3, 3/5, 26/45, 8/15, 1/2} [36,37]
	Baseline metric ( $\eta_{\mathrm{IR}}^{\left(\mathrm{base}\right)}$ )	1.08
	Total number of punctured and shortened bits (*n_d_*) [bit]	3200 for block length *N* 1200 for block length $N_{\mathrm{sb}}$
	Maximum number of decoding iterations [iteration]	100
	Increment factor for updating the efficiency metric (*δ*)	0.2
Subblock confirmation	Subblock size $(N_{\mathrm{sb}}$ ) [bit]	16,200
	Number of subblocks (*m*)	4
	Hash value length ( $l_{\mathrm{hash}})$ [bit /byte]	64/8

**Table 2:** Numerical results of random key sampling with 5% and 10% sampling rates and the syndrome estimation using *N* = 64,800 bits with $R_{C}^{\left(\max\right)}$ = 9/10 and $N_{\mathrm{sb}}$ = 16,200 bits with $R_{C}^{\left(\max\right)}$ = 8/9. The results are presented for the observed QBER values: (**a**) 2%, (**b**) 4%, (**c**) 7%, and (**d**) 10%.

**QBER Estimation Methods**	**5% Random** **Key Sampling**	**10% Random Key Sampling**	Syndrome est. (N = 64,800 bits/ $R_{C}^{\;(\max)}$ = 9/10)	Syndrome est. $(N_{\mathrm{sb}}$ = 16,200 Bits/ $R_{C}^{\;(\max)}$ = 8/9)
**(a) Observed QBER: 2%**				
Mean accuracy (%)	99.8092	99.8685	99.9545	99.9035
Mean estimated QBER (*q_est_*)	0.019962	0.020019	0.019994	0.020039
Mean squared error (MSE)	5.7996 × 10^−6^	2.7454 × 10^−6^	3.2805 × 10^−7^	1.4677 × 10^−6^
Median	0.020062	0.020062	0.019988	0.019978
Interquartile range (IQR)	0.003395	0.002315	0.001104	0.002225
Mean number of outliers	0.0090	0.0070	0.0065	0.0085
**(b) Observed QBER: 4%**				
Mean accuracy (%)	99.7352	99.8195	99.9220	99.8443
Mean estimated QBER (*q_est_*)	0.040069	0.040069	0.039987	0.040081
Mean squared error (MSE)	1.1270 × 10^−5^	5.1504 × 10^−6^	9.6916 × 10^−7^	3.8172 × 10^−6^
Median	0.040123	0.039969	0.039984	0.040027
Interquartile range (IQR)	0.004630	0.003241	0.001599	0.003413
Mean number of outliers	0.0115	0.0085	0.0120	0.0050
**(c) Observed QBER: 7%**				
Mean accuracy (%)	99.6501	99.7682	99.8574	99.7451
Mean estimated QBER (*q_est_*)	0.070037	0.070037	0.070031	0.070105
Mean squared error (MSE)	1.9198 × 10^−5^	8.4687 × 10^−6^	3.1647 × 10^−6^	1.0266 × 10^−5^
Median	0.070062	0.070062	0.069934	0.070078
Interquartile range (IQR)	0.006173	0.003858	0.002734	0.005078
Mean number of outliers	0.0060	0.0125	0.0080	0.0060
**(d) Observed QBER: 10%**				
Mean accuracy (%)	99.5781	99.7186	99.7808	99.6005
Mean estimated QBER (*q_est_*)	0.100013	0.099960	0.100085	0.100068
Mean squared error (MSE)	2.7643 × 10^−5^	1.2399 × 10^−5^	7.6506 × 10^−6^	2.4914 × 10^−5^
Median	0.100000	0.100000	0.100027	0.099976
Interquartile range (IQR)	0.007407	0.005093	0.003965	0.007457
Mean number of outliers	0.0070	0.0070	0.0120	0.0050

**Table 3:** Simulation results of random key sampling with 5% and 10% rates, compared with the syndrome estimation using *N* = 64,800 bits with $R_{C}^{\left(\max\right)}$ = 9/10 and $N_{\mathrm{sb}}$ = 16,200 bits with $R_{C}^{\left(\max\right)}$ = 8/9 in three observed QBER ranges: (**a**) low error range of 1.00–3.50%, (**b**) moderate error range of 3.51–7.00%, and (**c**) high error range of 7.01–11.00%.

**QBER Estimation Methods**	**5% Random** **Key Sampling**	**10% Random Key Sampling**	Syndrome est. (N = 64,800/ $R_{C}^{\;(\max)}$ = 9/10)	Syndrome est. $(N_{\mathrm{sb}}$ =16,200/ $R_{C}^{\;(\max)}$ = 8/9)
**(a) Low error range: observed QBER 1.00–3.50%**				
Mean accuracy (%)	99.7985	99.8618	99.9498	99.9006
Standard deviation (SD)	0.1589	0.1050	0.0417	0.0803
Lower error bar (mean—SD) (%)	99.6396	99.7568	99.9081	99.8203
Upper error bar (mean + SD) (%)	99.9574	99.9668	99.9915	99.9809
Mean squared error (MSE)	6.5846 × 10^−6^	3.0130 × 10^−6^	4.2539 × 10^−7^	1.6325 × 10^−6^
**(b) Middle error range: observed QBER 3.51–7.00%**				
Mean accuracy (%)	99.6941	99.7939	99.8984	99.8025
Standard deviation (SD)	0.2342	0.1609	0.0807	0.1545
Lower error bar (mean—SD) (%)	99.4599	99.6330	99.8177	99.6480
Upper error bar (mean + SD) (%)	99.9283	99.9548	99.9791	99.9570
Mean squared error (MSE)	1.4840 × 10^−5^	6.8321 × 10^−6^	1.6833 × 10^−6^	6.2887 × 10^−6^
**(c) High error range: observed QBER 7.01–11.00%**				
Mean accuracy (%)	99.6168	99.7283	99.8092	99.6556
Standard deviation (SD)	0.2935	0.2003	0.1490	0.2698
Lower error bar (mean—SD) (%)	99.3233	99.5280	99.6602	99.3858
Upper error bar (mean + SD) (%)	99.9103	99.9286	99.9582	99.9254
Mean squared error (MSE)	2.3293 × 10^−5^	1.1395 × 10^−5^	5.8601 × 10^−6^	1.9135 × 10^−5^
**Sifted key size after channel error estimation (bits)**				
*N* = 64,800/ $N_{\mathrm{sb}}$ = 16,200 bits (100% sifted key)	61,560/15,390 (95%)	58,320/14,580 (90%)	64,800 (100%)	16,200 (100%)

**Table 4:** Parameters of a BB84 QKD setup based on the inherent properties of the single photon source and detection, as well as the optical fiber losses of the quantum channel.

Parameter	Efficiency of BB84 Protocol ( $\varepsilon_{\mathrm{BB}84}$ )	Efficiency of Single-Photon Detector ( $\varepsilon_{\mathrm{detect}}$ )	Dark Count Probability of Single-Photon Detector (*p_dark_*)	Optical Fiber Losses (dB/km)
**Value**	0.5	0.1	10^−5^	0.2

## Data Availability

No new data were created or analyzed in this study. Data sharing is not applicable to this article.

## References

[1] Diffie W., Hellman M.E. (1976). New directions in cryptography. IEEE Trans. Inf. Theory..

[2] Rivest R., Shamir A., Adleman L. (1978). A method for obtaining digital signatures and public key cryptosystems. Commun. ACM.

[3] Strangio M.A. Efficient Diffie-Hellmann two-party key agreement protocols based on elliptic curves. Proceedings of the 20th Annual ACM symposium on Applied computing (SAC 2005).

[4] Arute F., Arya K., Babbush R., Bacon D., Bardin J.C., Barends R., Biswas R., Boixo S., Brandao F.G., Buell D.A. (2019). Quantum supremacy using a programmable superconducting processor. Nature..

[5] Yan B., Tan Z., Wei S., Jiang H., Wang W., Wang H., Luo L., Duan Q., Liu Y., Shi W. (2023). Factoring integers with sublinear resources on a superconducting quantum processor. arXiv.

[6] Bennett C.H., Brassard G. Quantum cryptography: Public key distribution and coin tossing. Proceedings of the IEEE International Conference on Computers, Systems, and Signal Processing.

[7] Renner R., Gisin N., Kraus B. (2005). Information-theoretic security proof for quantum-key-distribution protocols. Phys. Rev. A.

[8] Fung C.-H.F., Ma X., Chau H.F. (2010). Practical issues in quantum-key-distribution postprocessing. Phys. Rev. A.

[9] Kiktenko E.O., Trushechkin A.S., Kurochkin Y.V., Fedorov A.K. (2016). Post-processing procedure for industrial quantum key distribution systems. J. Phys. Conf. Ser..

[10] Brassard G., Salvail L. Secret-key reconciliation by public discussion. Proceedings of the Advances in Cryptology–EUROCRYPT ‘93, Workshop on the Theory and Application of Cryptographic Techniques.

[11] Pedersen T.B., Toyran M. (2015). High performance information reconciliation for QKD with cascade. Quantum Inf. Comput..

[12] Martinez-Mateo J., Pacher C., Peev M., Ciurana A., Martin V. (2015). Demystifying the information reconciliation protocol cascade. Quantum Inf. Comput..

[13] Buttler W.T., Lamoreaux S.K., Torgerson J.R., Nickel G.H., Donahue C.H., Peterson C.G. (2003). Fast, efficient error reconciliation for quantum cryptography. Phys. Rev. A.

[14] Makkaveev A.P., Molotkov S.N., Pomozov D.I., Timofeev A.V. (2005). Practical error-correction procedures in quantum cryptography. J. Exp. Theor. Phys..

[15] Treeviriyanupab P., Sangwongngam P., Sripimanwat K., Sangaroon O. BCH-based Slepian-Wolf coding with feedback syndrome decoding for quantum key reconciliation. Proceedings of the 9th International Conference on Electrical Engineering/Electronics, Computer, Telecommunications and Information Technology (ECTI-CON 2012).

[16] Pearson D. High-speed QKD reconciliation using forward error correction. Proceedings of the 7th International Conference on Quantum Communication, Measurement and Computing (QCMC 2004).

[17] Elkouss D., Leverrier A., Alléaume R., Boutros J.J. Efficient reconciliation protocol for discrete-variable quantum key distribution. Proceedings of the IEEE International Symposium on Information Theory (ISIT 2009).

[18] Martinez-Mateo J., Elkouss D., Martin V. (2012). Blind reconciliation. Quantum Inf. Comput..

[19] Liu Z., Wu Z., Huang A. (2020). Blind information reconciliation with variable step sizes for quantum key distribution. Sci. Rep..

[20] Kiktenko E.O., Trushechkin A.S., Lim C.C.W., Kurochkin Y.V., Fedorov A.K. (2017). Symmetric blind information reconciliation for quantum key distribution. Phys. Rev. Appl..

[21] Treeviriyanupab P., Phromsa-ard T., Zhang C.-M., Li M., Sangwongngam P., Sanevong Na Ayutaya T., Songneam N., Rattanatamma R., Ingkavet C., Sanor W. Rate-adaptive reconciliation and its estimator for quantum bit error rate. Proceedings of the 14th International Symposium on Communications and Information Technologies (ISCIT 2014).

[22] Kiktenko E.O., Malyshev A.O., Bozhedarov A.A., Pozhar N.O., Anufriev M.N., Fedorov A.K. (2018). Error estimation at the information reconciliation stage of quantum key distribution. J. Russ. Laser. Res..

[23] Gao C., Jiang D., Guo Y., Chen L. (2019). Multi-matrix error estimation and reconciliation for quantum key distribution. Opt. Express.

[24] Borisov N., Petrov I., Tayduganov A. (2023). Asymmetric adaptive LDPC-based information reconciliation for industrial quantum key distribution. Entropy.

[25] Fedorov A.K., Kiktenko E.O., Trushechkin A.S. (2018). Symmetric blind information reconciliation and hash-function-based verification for quantum key distribution. Lobachevskii J. Math..

[26] Krovetz T., Rogaway P. Fast universal hashing with small keys and no preprocessing: The PolyR construction. Proceedings of the 3rd International Conference on Information Security and Cryptology (ICISC 2000).

[27] Shannon C.E. (1948). A mathematical theory of communication. Bell Syst. Tech. J..

[28] Slepian D., Wolf J.K. (1973). Noiseless coding of correlated information sources. IEEE Trans. Inform. Theory.

[29] Gallager R. (1963). Low-Density Parity-Check Codes. Ph.D. Thesis.

[30] Tian T., Jones C.R. (2005). Construction of rate-compatible LDPC codes utilizing information shortening and parity puncturing. EURASIP J. Wirel. Comm..

[31] Elkouss D., Martinez-Mateo J., Martin V. (2011). Information reconciliation for quantum key distribution. Quantum Inf. Comput..

[32] Lo H.-K., Chau H.F., Ardehali M. (2004). Efficient quantum key distribution scheme and a proof of its unconditional security. J. Cryptol..

[33] Gottesman D., Lo H.-K., Lütkenhaus N., Preskill J. (2004). Security of quantum key distribution with imperfect devices. Quantum Inf. Comput..

[34] Ma X., Lütkenhaus N. (2012). Improved data post-processing in quantum key distribution and application to loss thresholds in device independent QKD. Quantum Inf. Comput..

[35] Martinez-Mateo J., Elkouss D., Martin V. (2013). Key reconciliation for high performance quantum key distribution. Sci. Rep..

[36] (2014). Digital Video Broadcasting (DVB); Second Generation Framing Structure, Channel Coding and Modulation Systems for Broadcasting, Interactive Services, News Gathering and Other Broadband Satellite Applications. Part 1: DVB-S2; (V1.4.1), 12 November 2014.

[37] (2021). Digital Video Broadcasting (DVB); Second Generation Framing Structure, Channel Coding and Modulation Systems for Broadcasting, Interactive Services, News Gathering and Other Broadband Satellite Applications. Part 2: DVB-S2 Extensions (DVB-S2X); (V1.3.1), 8 February 2021.

